# Supplementary material for: Information dynamics of in silico EEG Brain Waves: Insights into oscillations and functions
Source: PLoS Comput Biol. 2024 Sep 5;20(9):e1012369. doi: 10.1371/journal.pcbi.1012369 (PMC11407780; doi:10.1371/journal.pcbi.1012369)
Supplement: S1 Appendix — Contains complementary results and discussion presented in three sections: Power spectrum density across phase diagram, Phase diagram and rhythms and Changes of information measures with increasing time delay. (PDF) [file pcbi.1012369.s001.pdf]

Information dynamics of *in silico* EEG Brain Waves: Insights into oscillations and functions

Gustavo Menesse Joaquín J. Torres

August 18, 2024

1 Power spectrum density across phase diagram

Fig A shows the maxima in the power spectrum for each band in the noise level vs synaptic resources time constant parameter space (μ, τ_{rec}). Several interesting behaviors can be observed, such as the modulation of β rhythms by δ waves in the meta-stable region (between the dashed white lines) and the emergence of low-frequency rhythms (δ, θ, α) in a region of high inhibitory activity, which occurs between the LAI-like phase transition of both populations (between the dashed-dotted and dotted white lines). This last phenomenon were not explore in the current article.

Fig B show the average membrane potential of five groups of excitatory (A) and inhibitory (B) neurons at different points in the parameter space (μ, τ_{rec}). A notable observation is that high-frequency oscillations (γ_{fast}) in excitatory neurons are less noisy compared to those in the inhibitory population. This is related to lower mutual information values in the inhibitory population in regions where inhibitory γ_{fast} oscillations emerge (see main text Discussion about high frequency oscillations regimes in this model).

The full power spectrum densities for different values of τ_{rec} with fixed μ are shown in Fig C. Similarly, for different values of μ with fixed τ_{rec}, the power spectrum densities are displayed in Fig D. In these figures, a moving average is applied to the power spectrum between frequencies 0 and 100 Hz to enhance the visualization of the different curves. However, this averaging modifies the power intensities, causing the values in these plots to not match the maximum values shown in the earlier figures. Nonetheless, the important details and order relations between band intensities still hold.

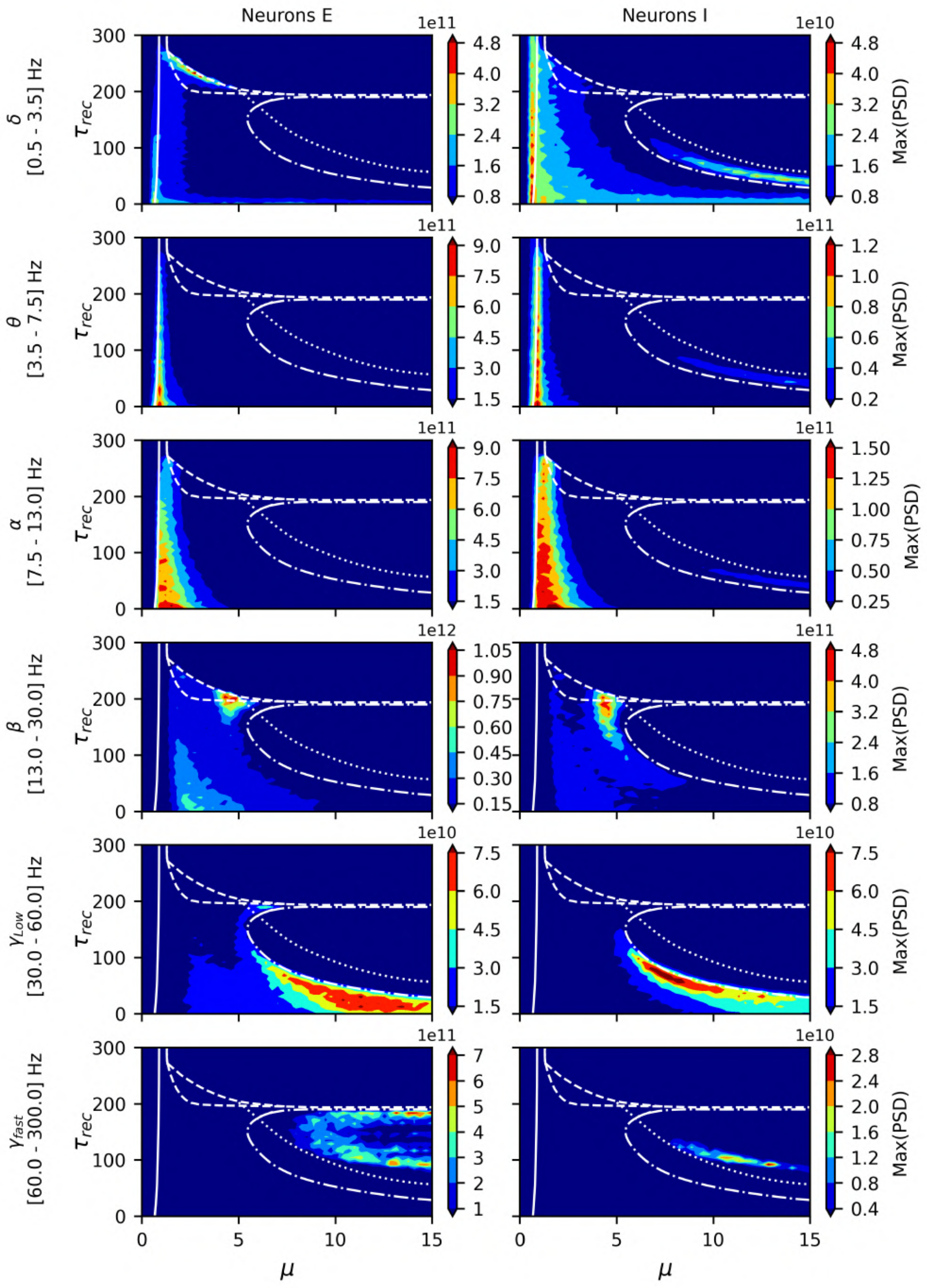

Figure A: **Maximum of power spectral density (PSD) for given frequency bands (rows) across the phase diagram (μ, τ_{rec}) for excitatory neurons (left panels) and inhibitory neurons (right panels)**. PSD was calculated from the average membrane potential of a group of excitatory and inhibitory neurons (see Fig 4A of main text). The dotted (dash-dotted) line indicates the maximum variance of the excitatory (inhibitory) neurons state X. The solid (dashed) white lines indicate a second-order (first-order) phase transition. Low frequencies dominate in the low noise region after the second-order transition ($0.5 \lessapprox \mu \lessapprox 4$) (see rows 1, 2 and 3). However, excitatory δ waves are strongest close to the first order transition in the meta-stable region (between white dashed lines). On the other hand, β waves are also stronger in the metastable region for higher noise values ($4.5 \lessapprox \mu \lessapprox 6.5$), but extend their dominance for lower τ_{rec}, below the meta-stable region (see row 4). Moreover, γ_{Low} dominates in the high-noise and low τ_{rec} region, close to the maximum variance of inhibitory neuron states X (dashed-dotted white lines) (see row 5) . Finally, high frequencies rhythms emerge in the E-I high-activity region (region enclosed by the dotted white curve) (see row 6). High-frequency waves (γ_{fast}) have a complex pattern similar to that observed for the variance of inhibitory activity (see Fig 5B bottom of main text), indicating that these rhythms could be related to complex activity in the inhibitory population.

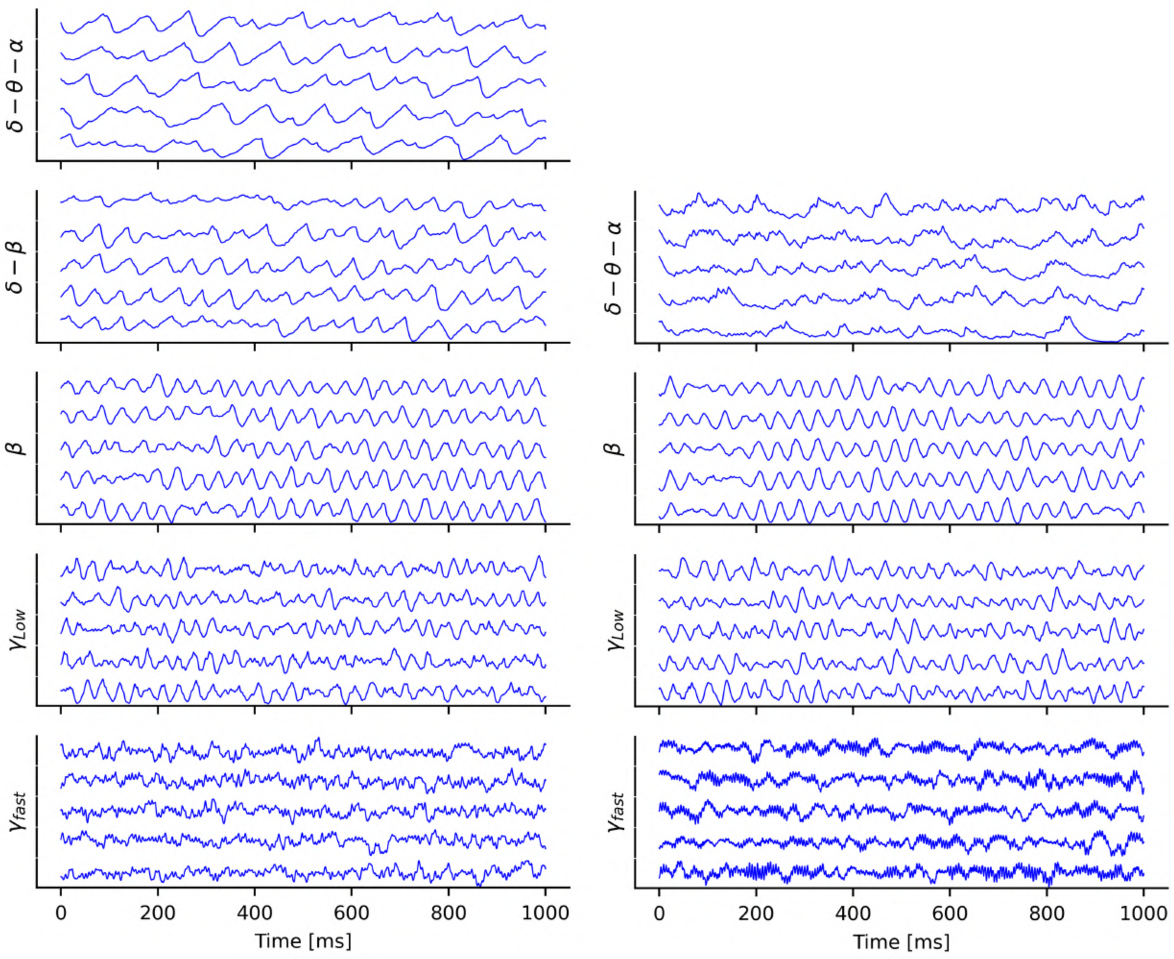

(A)

(B)

Figure B: **Time series of average membrane potential of excitatory neurons (Column A, left) and inhibitory neurons (Column B, right) in different points of parameter** (μ, τ_{rrec}) **space (rows)**. Each panel shows times series correspond to average of 5 group of neurons for each population as indicated in the Material and methods section (Fig 4A). Each row shows the time series related to higher PSD values for different waves as indicated in the y-axis label of each panel. Points used are shown as colored stars in Fig 6A and Fig 6B.

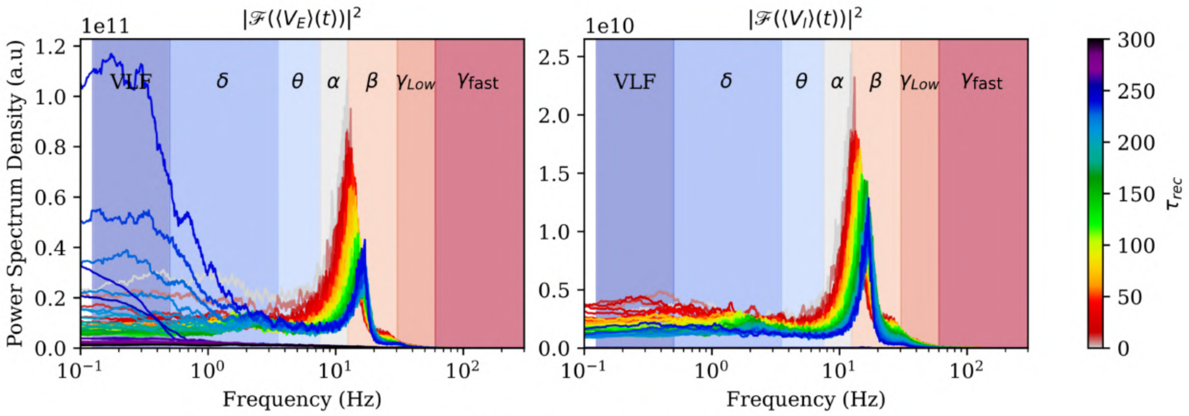

(A) Fixed μ = 2.499

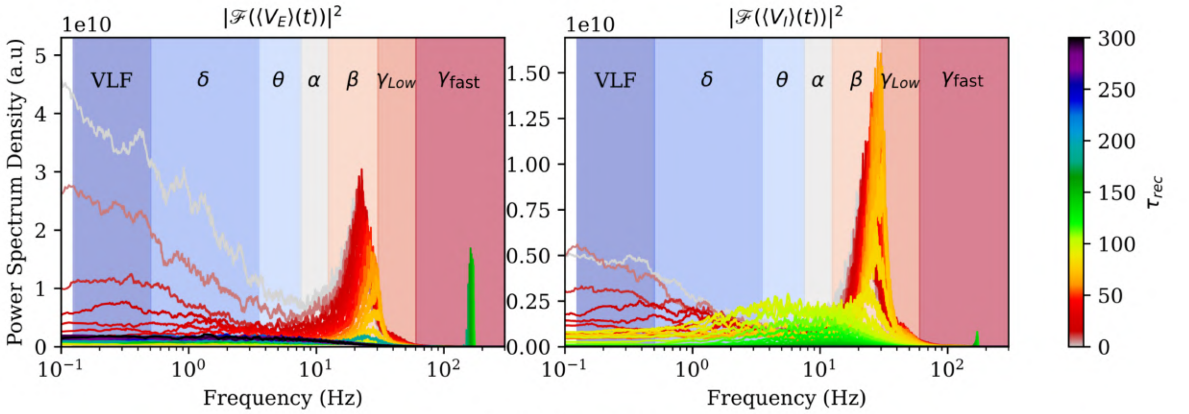

(B) Fixed μ = 7.633

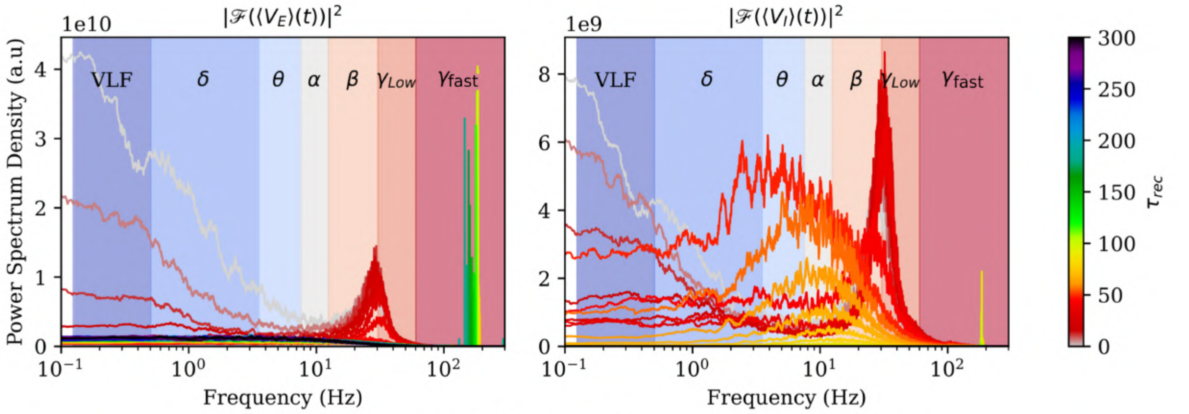

(C) Fixed μ = 12.551

Figure C: **Full power spectral density (PSD) of average membrane potential in a group of neurons vs τ_{rec} for fixed values of $\mu = \{2.449, 7.653, 12.551\}$ in Panels (A), (B) and (C), respectively.** (Left A-C) PSD of average excitatory membrane potential. (Right A-C) PSD of average inhibitory membrane potential. Color code indicates the value of synaptic recovery time constant τ_{rec} as shown in the color bar of each panel. Waves can be observed until the first-order phase transition ($\tau_{rec} \approx 200$). Wave frequency increases with increasing external noise. In panel (C), we see that γ waves have high PSD only in a narrow band, first appearing at $\tau_{rec} \approx 90$ ms (yellow). In excitatory neurons (left panel), the waves disappear for $140 \lesssim \tau_{rec} \lesssim 160$ ms, reappearing after $\tau_{rec} = 160$ ms (green) but with a lower frequency, probably caused by the reduction in the available synaptic resources as τ_{rec} increases. In the inhibitory neuron group, the waves are noisier with increasing values of μ (less smooth curves). This shows how inhibition stabilizes the activity in the exitatory population, but there is no stabilization in the inhibitory population, because the model does not include I-I connections.

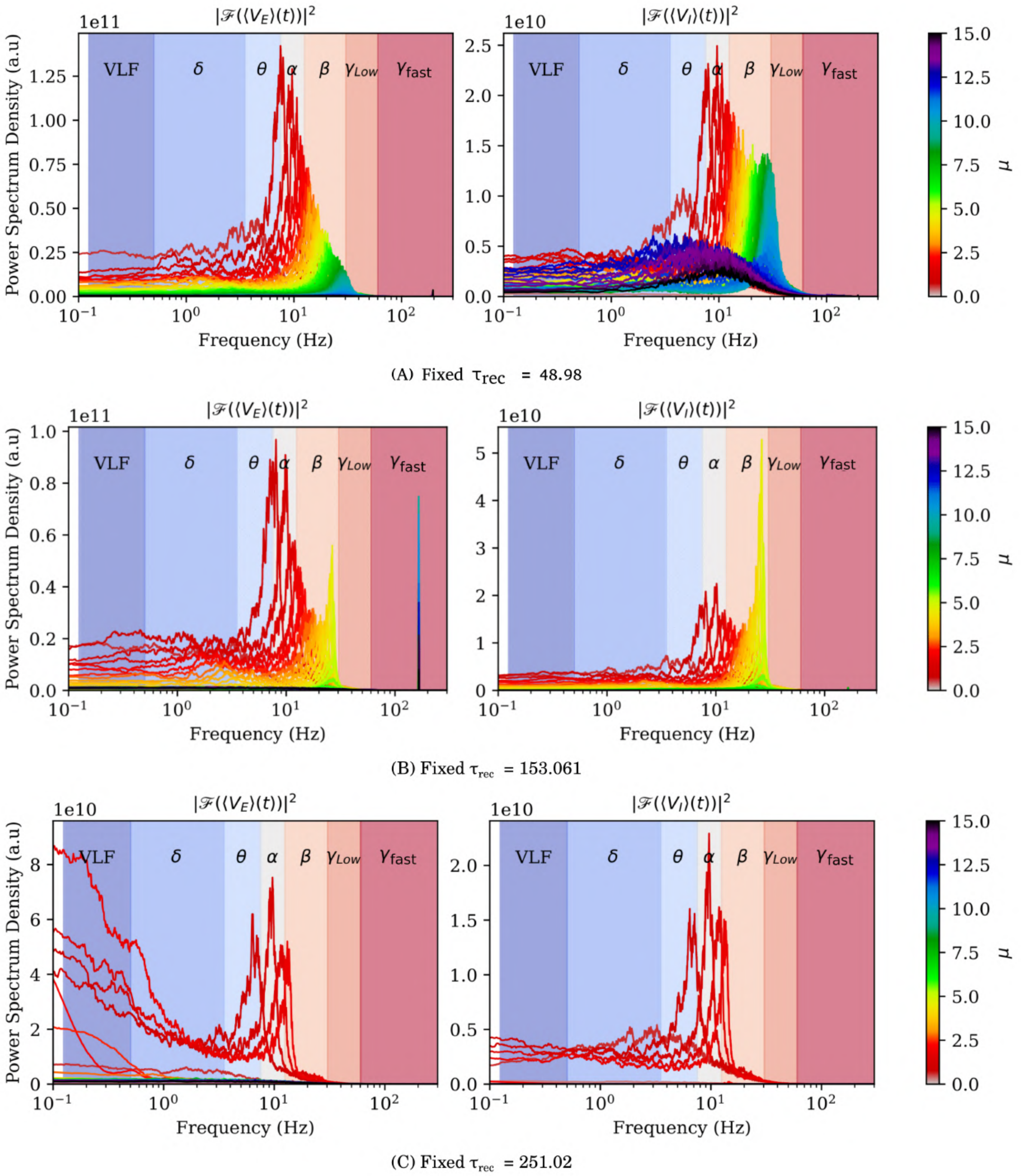

Figure D: **Full power spectral density (PSD) of average membrane potential in a group of neurons vs μ for fixed values of $\tau_{rec} = \{48.98, 153.061, 251.02\}$ ms in Panels (A), (B) and (C), respectively**. (Left A-C) PSD of average excitatory membrane potential. (Right A-C) PSD of average inhibitory membrane potential. Color code indicates the value of noise level μ as shown in the color bar of each panel. In Panel (A), for low τ_{rec}, the amplitude of the waves decreases with increasing noise. In the excitatory neurons, the frequency of dominant wave increases monotonically with noise level. In Panel (B), the amplitude first decreases with noise, but a peak of amplitude is observed for $2.5 < \mu < 5$ in the high β or almost γ_{Low}. In the inhibitory population, this high β peak is higher, being almost 3 times larger than the amplitudes of other waves. In Panel (C), for $\tau_{rec} > 200$ ms, waves emerge only in a narrow band of μ values, corresponding to the phase III, and close to the continuous phase transition between phase I and III (see Fig 5 of main text).

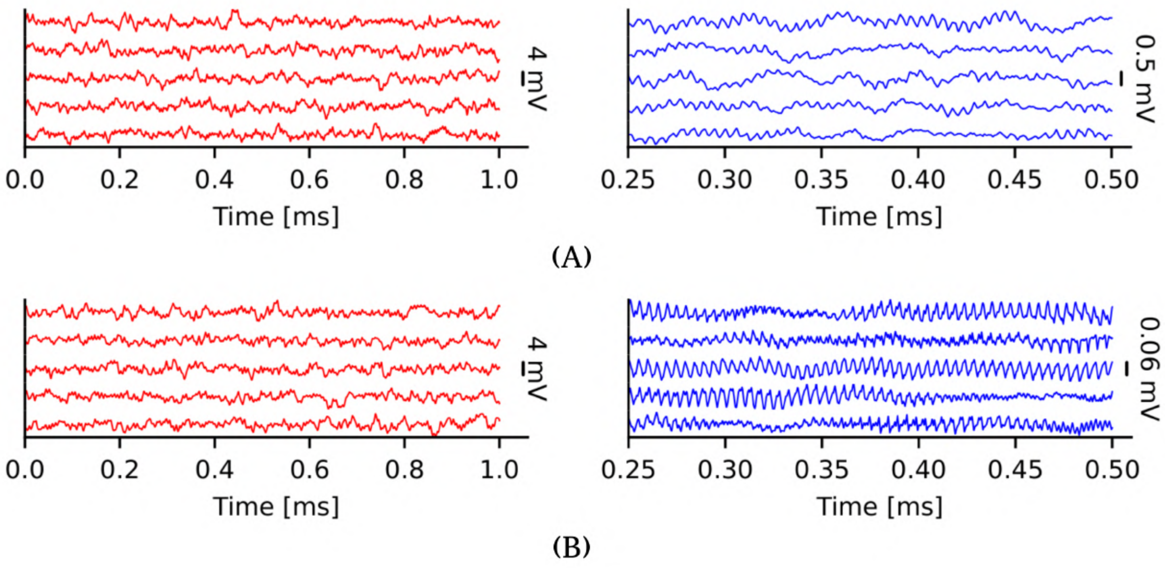

Figure E: **Emergence of γ_{fast} in average membrane potential have two different regimes.** All panels depict time series of average membrane potential in 5 excitatory (red) and inhibitory (blue) neuron groups at points of maximum PSD of excitatory γ_{fast} (top panel A) and inhibitory γ_{fast} (bottom panel B). The five time series of each panel correspond to each five group of neurons (see groups in Fig 4A of Material and methods in main text). (Top panel A) Regime 1: high amplitude only in excitatory neurons. Fast γ band have its higher PSD maximum in excitatory neuronal population, while the amplitude of the oscillations in the inhibitory population are tiny (less than 0.1 mV, note that we have amplify the amplitude for a better visualization), and therefore, we do not consider it as relevant rhythm. (Bottom panel B) Regime 2: high amplitude in both neuronal population. Coexistence of excitatory and inhibitory rhythms in the fast γ band at the point of the parameter space (μ, τ_{rec}) of higher PSD maximum in the inhibitory population. Here, the amplitude of inhibitory oscillations if 10 times larger than in Regime 1 (Panel A), therefore, not negligible.

2 Phase diagram and rhythms

2.1 High frequency oscillations in E-I high activity phase.

Fast γ oscillations (> 80 Hz) emerge in the high activity phase in both neuronal populations, but inhibitory oscillations are only dominant (PSD $> 10^{10}$) in a restricted area, where they coexist with excitatory γ rhythms, just above the LAI phase transition in the excitatory population, in the so-called high E-I activity phase (II.b).

To exemplify the emergence of high-frequency oscillations in the average membrane potential in our system, Fig E shows a 1-second time window of the average membrane potential of 5 excitatory and inhibitory neuron groups at points in the parameter space where excitatory and inhibitory γ_{fast} waves have their maximum peak. This time series demonstrates how we can generate em in silico with this model a complex signal that closely resembles actual EEG and LFP data. The power spectrum of these time series is shown in Fig F.

Although these *in silico* complex generated rhythms also include the possibility of the coexistence of different rhythms over time, in this section of Supplementary Material we will focus on explaining the different behaviors observed in the region of dominant fast γ oscillations, as this can be connected with current discussions about the nature and meaning of HFOs.

2.2 Neural activity and emergence of HFO's.

We observed no clear relation between neuronal activity ρ and the emergence of high-frequency oscillations (see Fig G panels B and E), since there are points where excitatory activity is high but no dominant peak is observed in PSD. In fact, the values with maximum inhibitory activity appear to have a lower power (maximum of the PSD less than 10^{11} for E and less than 2×10^{9} for I), indicating a non-linear

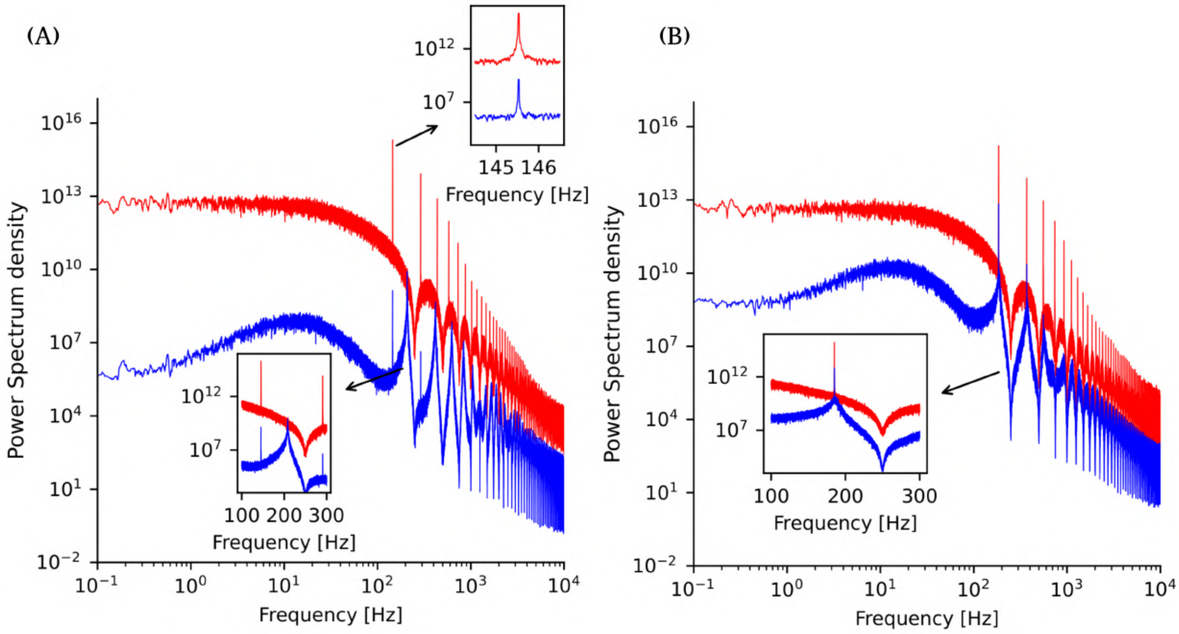

Figure F: **Power spectrum density (PSD) of average membrane potential time serie**. (A) PSD of excitatory (red) and inhibitory (blue) neuron groups at the point of maximum PSD in the excitatory fast γ band. The figure depicts dominant oscillation frequency of the excitatory group around 145.5 Hz, while the inhibitory group shows low power oscillations at 200 Hz, which corresponds to the low amplitude inhibitory oscillations shown in Fig E panel A. A smaller peak of inhibitory oscillations is observed at 145.5Hz, seemingly induced by excitatory oscillations at the same frequency. (B) PSD of excitatory (red) and inhibitory (blue) neuron groups at the point of maximum PSD in the inhibitory fast γ band. Where excitatory and inhibitory oscillations coexist, both populations have a high power peak at the same frequency, close to 190 Hz. This frequency synchronization appears to be responsible for the increase in inhibitory oscillation amplitude shown in Fig E panel B, where the amplitudes are almost 10 times larger than in the Fig E panel A.

relationship between the emergence of γ_{fast} and neuronal activity. However, we observed a minimum inhibitory activity necessary for the occurrence of γ_{fast} waves, since all points with maximum of PSD larger than 10 for excitatory and larger than 9 for inhibitory have $\rho^I \gtrapprox 0.55$. Therefore, it is clear that the emergence of these rhythms in our system is related with the inhibitory activity, although increasing inhibitory activity does not explain in general the increase in PSD peak, as can be seen in the same figures.

Comparing the variance of the neuronal activity with the maxima of PSD for γ_{fast} waves (Fig G panels C and F), we see that the excitatory fast γ rhythms have a larger power when inhibitory activity has a high temporal variance in a specific interval of inhibitory activity (i.e. $0.6 < \rho^I < 0.72$). On the contrary, the emergence of inhibitory fast γ oscillations is not related to the temporal variance of the excitatory activity in a similar way. As we can see in Fig G panel F, there is a set of points where the PSD peak increases even with decreasing variance and average of the excitatory activity. Thus, examining only the variance of temporal activity is insufficient to understand the emergence of these rhythms.

In conclusion, our analysis illustrates that the emergence of fast γ rhythms is not merely a consequence of high firing rates. While the time variance of activity in one population appears to be related to rhythm emergence in the other population, there are exceptions, indicating that this relationship is not universal. Despite the simplicity of the present model, rhythms emerge from a complex interplay between excitatory and inhibitory populations. Global variables such as average neuronal activity (the typical mean-field observable used in the statistical mechanics approach) are not sufficient to describe/explain this complexity.

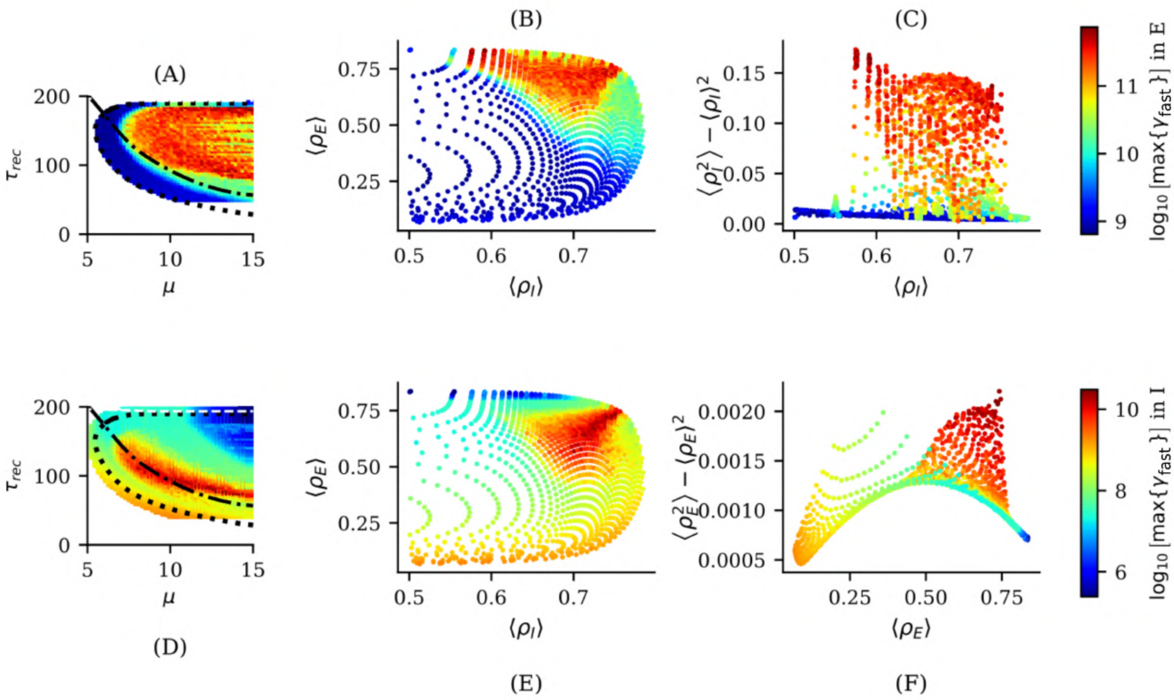

Figure G: **Emergence of γ_{fast} is related to the fluctuations in inhibitory activity**. Color code indicate the $\log_{10}$ of maximum power spectrum density (PSD) in the γ_{fast} band. Top: (A) maximum γ_{fast} PSD of excitatory population at points of the space parameter (μ, τ_{rec}) where $\rho_{E/I} > 0.5$ (Phase II.b); (B) scatter plot of average acivity in inhibitory (x-axis) vs excitatory (y-axis) neuronal populations in phase II.b; (C) scatter plot of temporal variance of activity vs average activity in inhibitory population. Bottom: (D) maximum γ_{fast} PSD of inhibitory population at points of the space parameter (μ, τ_{rec}) in Phase II.b; (E) scatter plot of temporal average inhibitory (x-axis) vs excitatory (y-axis) activity in phase II.b; (F) scatter plot of temporal variance of activity vs average activity in excitatory population. In panel (B) we also observe that fixing a ρ^E or ρ^I value above 0.5, γ_{fast} PSD could almost cover the full extend of values, indicating there is no direct relation between activity and the emergence of high frequency oscillations. This observation demonstrates that this rhythm is not a simple effect of high firing rate caused by a random external input. On the other hand, panel (C) also illustrates that γ_{fast} PSD in the excitatory population increases with the variance in inhibitory activity. This suggests that in our system, excitatory γ_H rhythms are related to the interaction between excitatory activity and fluctuations in inhibitory activity. Finally, panel (F) illustrates that the variance of excitatory activity is related to higher γ_{fast} PSD in inhibitory population, but with exceptions. A cloud of points of intermediate and high power emerges even with low excitatory activity and variance, suggesting a complex relation between inhibitory waves and excitatory activity.

3 Changes in information measures with increasing time delay

In this section, we illustrate how integrated information, redundant information, and differentiated information in both excitatory and inhibitory neurons evolve with varying time delays between the current state $\mathbf{X}[t]$ and future state $\mathbf{X}[t+\tau]$. This analysis provides a comprehensive view of the information dynamics time scale for each neuronal population. Figs H and I depict the information measures computed with time delays $\tau = 10$ time bins (40 ms) and $\tau = 100$ time bins (400 ms) for both neuronal populations.

Two main observations arise:

- Integrated information (Φ^R) exhibits time delay-invariance during the low activity intermediate (LAI) phase transition.
- Redundant and differentiated information ($\mathcal{R}$ and $\mathcal{U}$, respectively) decay with increasing time delay in phases II.a and II.b of the excitatory population. However, these measures display a complex pattern in the same phase (II.b) for the inhibitory neurons.

For a detailed discussion on the implications of these observations, please refer to the Discussion section in the main text.

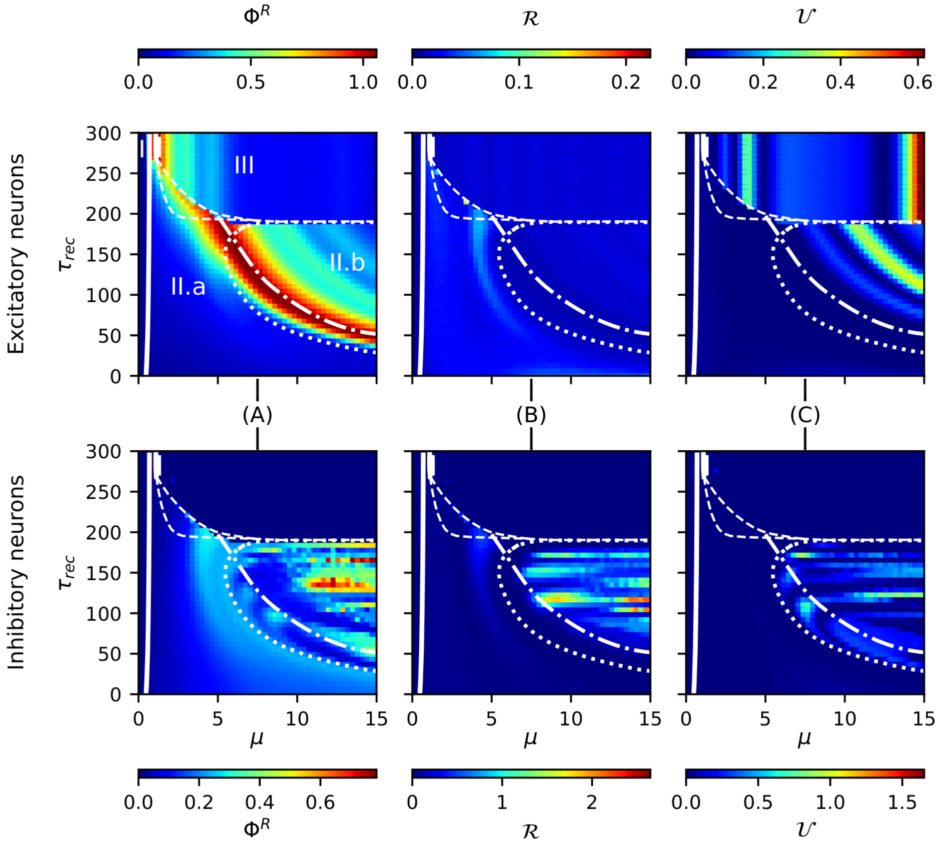

Figure H: **Information measures (Φ^R, $\mathcal{R}$, $\mathcal{U}$) for delay $\tau = 10$ time bins (40 ms)**. (A, top and bottom) Integrated information in both excitatory and inhibitory group has the same behavior as that observed for time delayed $\tau = 1$ time bin (4 ms) (see Fig 7 of main text). A maximum in the transition between LAI phase and high activity phase in excitatory population and spots of high integrated information in high inhibitory activity (see main text) are depicted. (B-C, top and bottom) Redundant and Differentiated information change considerably with respect to the measures done with $\tau = 1$ bin (4 ms) (see Fig 7 of main text). The differentiated information in excitatory population has clear bands of high and low values dependent on noise level, which is the effect of a high variance in the individual neuron inter-spike interval for the binned time series (not shown). In phase III all excitatory neuron are decoupled, because there is no inhibitory activity. Therefore, all information we can have about the future of the system is unique to each neuron, which explain that the only measure that show high information is the differentiated information $\mathcal{U}$.

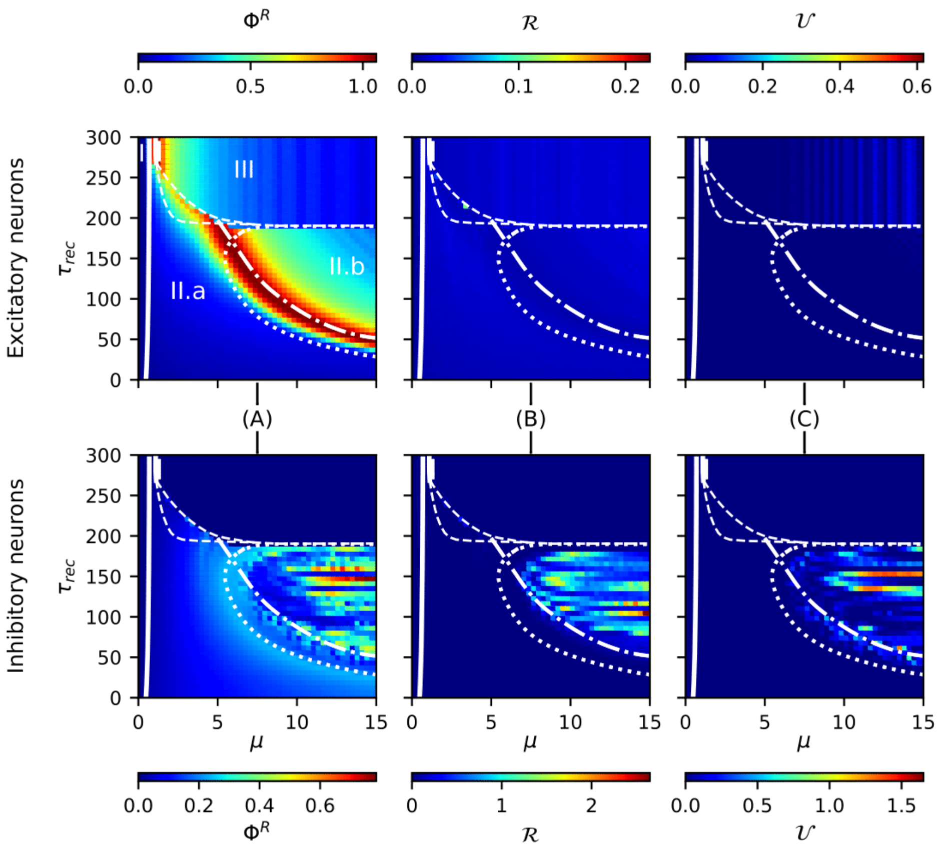

Figure I: **Information measures (Φ^R, $\mathcal{R}$, $\mathcal{U}$) for $\tau = 100$ time bins (400 ms).** (A, top and bottom) Integrated information presents the same behavior as that observed for time delays $\tau = 1$ and $\tau = 10$ bins. In the excitatory case, Φ^R seems to be time-invariant where it reaches maximum, which coincides with the maximum excitatory state variance line (dashed dotted white line) that marks the transition between the LAI phase and the high activity phase. (B-C) Redundancy and differentiation decrease considerably in the excitatory population compared to the measures for delays of $\tau = 1$ and $\tau = 10$ time bins, while maintaining high values in the inhibitory high activity phase. The information dynamics in the inhibitory population shows informational measure patterns in the parameter space that have a complex dependence on the time delay τ. In the excitatory population, however, the information dynamics decrease, in general, with time delay with the exception of the transition region, where Φ^R seems to be invariant with time.
