# Supplementary material for: Information dynamics of in silico EEG Brain Waves: Insights into oscillations and functions
Source: PLoS Comput Biol. 2024 Sep 5;20(9):e1012369. doi: 10.1371/journal.pcbi.1012369 (PMC11407780; doi:10.1371/journal.pcbi.1012369)
Supplement: Attachment — Submitted filename: Review_PLoSCB.pdf [file pcbi.1012369.s002.pdf]

The manuscript presents a review of information dynamics analyses of neural oscillatory signals and novel perspectives about information theoretical metrics that could be used to investigate the complex interactions between multivariate neural signals. Although the overall perspective and approach are interesting, the degree of novelty is somehow limited. Here is a detailed review of the major issues I observed in the manuscript.

ARTICLE TYPE

In my opinion, this may limit the impact of the manuscript. Overall, it seems to me that the manuscript presents a novel perspective, rather than original results that could be presented in a full Research Article. In fact, the manuscripts states the "our work here is an example of how information dynamics analysis through tools such as Φ-ID can provide insight about the emerging behavior, capturing details about dynamical phases and collective phenomena, which are complementary to those acquired by tools of statistical physics and nonlinear dynamics". Or again: "From the neuroscience point of view, an analysis like the one done here, when applied to experimental data in combination with models, could help researchers differentiate brain states, regions, and functions". This seems to fit more a Perspective paper to me. I leave this decision to the Editors.

INTRODUCTION

As far as I understand, the aim of the paper is to try to cover and link three main fields of research and associated literature: i) EEG brain rhythms and model; ii) information dynamics, PID and Integrated information decomposition (Φ-ID) framework; iii) HFOs. The link between i) and ii) is surely interesting and needs further study. And this manuscript is surely a nice example of the potential exploration in this regard. On the other hand, it is not clear to me why the manuscript tries to bridge the gap with the HFO literature. In fact, the model presented and used in study is not a specific for HFOs, but it produces a large panel of oscillatory states in different frequency bands, such as the alpha, beta, etc. I think the manuscript would benefit in clarity if the HFO part would be moved to the discussion and that the HFO part would be considered as a "Use case" of the model and information dynamics approach. The introduction would become shorter and the aim of the manuscript would become more evident, as far as I am concerned.

METHODS

The methods present very clearly the two main axes of the study: computational model of neural oscillations and the integrated information framework. As far as I understand, both have been published in previous papers. It is not clear to me whether some novel proposals are present in the study. The manuscript should state more clearly such novelty points. For example, one of the three information theoretical metrics have been proposed in the past already (effective information), and it is not clear whether the other two metrics are original.

RESULTS

Figure 5 to 9 present the analysis of the simulated time series. It is not clear which results are novel with respect to previous studies of such model. The manuscript should state more clearly

what are the novel aspects of the analyses of the simulations. If these results have been published in previous papers, I would suggest removing all these figures or reducing them significantly.

The "use case" on HFO is surely interesting and I think it represents the main result of the study. If the previous figures about the model states are not novel, I would suggest to restrain the focus of the results on the figures showing the information dynamics results on simulated data.

I suggest moving the section "Limitations of our analysis from experimental point of view" to a separate section. I think the Discussion and Results should be separated for clarity.

DATA AND CODE AVAILABILITY

Since one of the aims of the study is to provide a framework that could be used on neuroscientific experimental data, the manuscript should provide the underlying code. More generally, it is good practice for this type of study to share the data and code. As far as I understand, the Data and Code Availability section is missing.

SUPPLEMENTARY

I think the supplementary data is too extensive and it could be reduced or summarised. The readability of the paper would benefit.

REFERENCES

Several recent references applying information dynamics approaches to simulated or neural data are missing. In particular, I would suggest to check these:

https://www.pnas.org/doi/abs/10.1073/pnas.2300888120

https://www.nature.com/articles/s42003-023-04843-w

https://www.pnas.org/doi/abs/10.1073/pnas.2207677120

https://www.jneurosci.org/content/early/2023/11/13/JNEUROSCI.0157-23.2023.abstract

CONCLUSION

Overall, I think that the manuscript covers a very timely and interesting topic. Nevertheless the structure and focusing of the paper should be significantly revised. I also suggest considering a Perspective paper rather than a Research article.

Best regards.
