# Supplementary material for: Information dynamics of in silico EEG Brain Waves: Insights into oscillations and functions
Source: PLoS Comput Biol. 2024 Sep 5;20(9):e1012369. doi: 10.1371/journal.pcbi.1012369 (PMC11407780; doi:10.1371/journal.pcbi.1012369)
Supplement: Attachment — Submitted filename: Letter_to_Reviewers.pdf [file pcbi.1012369.s003.pdf]

Reviewer #1:

The manuscript presents a review of information dynamics analyses of neural oscillatory signals and novel perspectives about information theoretical metrics that could be used to investigate the complex interactions between multivariate neural signals. Although the overall perspective and approach are interesting, the degree of novelty is somehow limited. Here is a detailed review of the major issues I observed in the manuscript.

ARTICLE TYPE

Comment 1. In my opinion, this may limit the impact of the manuscript. Overall, it seems to me that the manuscript presents a novel perspective, rather than original results that could be presented in a full Research Article. In fact, the manuscripts states the "our work here is an example of how information dynamics analysis through tools such as Φ-ID can provide insight about the emerging behavior, capturing details about dynamical phases and collective phenomena, which are complementary to those acquired by tools of statistical physics and nonlinear dynamics". Or again: "From the neuroscience point of view, an analysis like the one done here, when applied to experimental data in combination with models, could help researchers differentiate brain states, regions, and functions". This seems to fit more a Perspective paper to me. I leave this decision to the Editors.

We respectfully disagree with the reviewer's point of view. The detailed exploration of the phase diagram of this model and the comprehensive analysis presented here are novel contributions. Our results represent a significant extension of the existing literature on this model.

However, we acknowledge that some statements in the manuscript may have conveyed a "Perspective" tone, suggesting how this new information theory tool can complement more traditional tools in statistical physics and nonlinear dynamics. To address this, we have revised these sentences to more clearly emphasize the originality and significance of our findings.

INTRODUCTION

Comment 2. As far as I understand, the aim of the paper is to try to cover and link three main fields of research and associated literature: i) EEG brain rhythms and model; ii) information dynamics, PID and Integrated information decomposition (Φ-ID) framework; iii) HFOs. The link between i) and ii) is surely interesting and needs further study. And this manuscript is surely a nice example of the potential exploration in this regard. On the other hand, it is not clear to me why the manuscript tries to bridge the gap with the HFO literature. In fact, the model presented and used in study is not specific for HFOs, but it produces a large panel of oscillatory states in different frequency bands, such as the alpha, beta, etc. I think the manuscript would benefit in clarity if the HFO part would be moved to the discussion and that the HFO part would be considered as a "Use case" of the model and information dynamics approach. The introduction would become shorter and the aim of the manuscript would become more evident, as far as I am concerned.

We agree with the reviewer. Following the comments of both reviewers, in the revised version of the manuscript we have decided to focus on the relation between EEG and Information Dynamics in general within the present EEG model and to use the discussion on HFOs only as one of the study cases. Additionally, we now also focus on the informational properties associated with other waves, such as beta and delta, in both excitatory and inhibitory neurons [See new Results subsections]. Experimental tests for our hypothesis about the informational properties of pathological and physiological HFOs will be stated as future directions, given the speculative nature of this topic.

METHODS

Comment 3. The methods present very clearly the two main axes of the study: computational model of neural oscillations and the integrated information framework. As far as I understand, both have been published in previous papers. It is not clear to me whether some novel proposals are present in the study. The manuscript should state more clearly such novelty points. For example, one of the three information theoretical metrics have been proposed in the past already (effective information), and it is not clear whether the other two metrics are original.

> *We appreciate the reviewer's insightful comments. In the revised manuscript, we have added clear statements delineating which metrics are novel and which have been previously proposed. As the reviewer notes, "effective information" and the definition of integrated information are not original, and we have clearly cited the sources to acknowledge this.*
>
> *However, the definitions proposed for Differentiated (U) and Redundant (R) information are novel and original contributions. These metrics are inspired by the strategy used to define Integrated Information, but substitute the effective information with new measures to capture unique and redundant information, respectively.*
>
> *Additionally, the new version of the manuscript includes other measures, such as information transfer and information storage, which are not original [see the new first subsection of Material and Methods]. We use these measures to characterize regions of our model's phase diagram where different oscillatory states, such as beta and delta waves, emerge. This shift in focus allows us to address wave emergence in general, not just HFOs as in the previous version of the manuscript.*

RESULTS

Comment 4. Figure 5 to 9 present the analysis of the simulated time series. It is not clear which results are novel with respect to previous studies of such model. The manuscript should state more clearly what are the novel aspects of the analyses of the simulations. If these results have been published in previous papers, I would suggest removing all these figures or reducing them significantly.

> *In the revised version of the manuscript, we have added new text to clarify the novel and original aspects of the analyses performed in the present work and highlighted the figures that present results not included in previous studies. We would like to inform the reviewer that all figures present some information that is not described in previous literature.*
>
> *While the main phases of this model (I, II, III, and a metastable phase) were already identified previously, the behavior of activity in excitatory and inhibitory populations was not explored in earlier works. Therefore, we consider it necessary to include new phase diagrams showing distinctions, such as different behaviors appearing in phase II. Additionally, the phase diagrams showing the regions where different waves dominate and the phase diagrams of information metrics are new and relevant for the present manuscript.*

Comment 5. The “use case” on HFO is surely interesting and I think it represents the main result of the study. If the previous figures about the model states are not novel, I would suggest to restrain the focus of the results on the figures showing the information dynamics results on simulated data.

As explained before, all results that describe in detail the phase diagram for this model are in fact novel and original and not reported yet elsewhere. As stated above and following the suggestion of the reviewer, the analysis and study of HFO have now been considered as an "use case" in the new version of the manuscript. Also we have focused the main results of the manuscript on the information dynamics of all emerging rhythms appearing throughout the whole phase diagram of the system, not only on HFO, following the second reviewer suggestion.

Comment 6. I suggest moving the section "Limitations of our analysis from experimental point of view" to a separate section. I think the Discussion and Results should be separated for clarity.

We agree with the reviewer's suggestion. In the revised version of our manuscript, we have separated the Discussion and Results sections and moved the section "Limitations…" to a separate section.

DATA AND CODE AVAILABILITY

Comment 7. Since one of the aims of the study is to provide a framework that could be used on neuroscientific experimental data, the manuscript should provide the underlying code. More generally, it is good practice for this type of study to share the data and code. As far as I understand, the Data and Code Availability section is missing.

We appreciate the reviewer's suggestion. In the revised version of the manuscript, we have included a Data and Code Availability section to provide access to the underlying code used in our study. We recognize the importance of transparency and reproducibility in scientific research and are committed to sharing our data and code to facilitate further exploration and validation of our findings.

SUPPLEMENTARY

Comment 8. I think the supplementary data is too extensive and it could be reduced or summarized. The readability of the paper would benefit.

We agree with the reviewer's suggestion, and in the revised version of the manuscript, we have reduced the supplementary data to improve readability without compromising the integrity or clarity of the study.

REFERENCES

Comment 9. Several recent references applying information dynamics approaches to simulated or neural data are missing. In particular, I would suggest to check these:
https://www.pnas.org/doi/abs/10.1073/pnas.2300888120
https://www.nature.com/articles/s42003-023-04843-w
https://www.pnas.org/doi/abs/10.1073/pnas.2207677120
https://www.jneurosci.org/content/early/2023/11/13/JNEUROSCI.0157-23.2023.abstract

Thanks to the reviewer for highlighting these important references. In our revised version, we have included and discussed some of these references in the Introduction section, which enrich the discussion and provide valuable context for our study.

CONCLUSION: Overall, I think that the manuscript covers a very timely and interesting topic. Nevertheless the structure and focusing of the paper should be significantly revised. I also suggest considering a Perspective paper rather than a Research article.

Best regards.

> *We are grateful to the reviewer for their positive assessment of the research conducted in our manuscript. We have diligently revised the manuscript in accordance with their comments and suggestions.*

Reviewer #2:

Comment 0: This is potentially an interesting work on the intersection of EEG network modeling, the role of oscillatory activity in information processing and application of current method of analysis to shed light on information dynamic in multivariate system. In this paper the authors present the phase diagram of the simulated EEG model to characterize different regimes related to the network activity. Subsequently, the authors show how different rhythms emerge in the phase diagram and provide an information dynamic analysis. High-gamma rhythms emerge in different neuronal populations (excitatory or inhibitory) with different information processing characteristics.

> *Thanks to the reviewer for their positive assessment of our manuscript.*

Commnet 1: The underlying concept of the manuscript, as presented in the abstract and introduction, specifically pertains to using a model to gain insights into the information processing characteristics of low/high gamma oscillations. This aims to enhance our understanding and ability to distinguish between pathological oscillatory activity and that related to cognitive functions. However, the manuscript devotes only a final part to the role of high-frequency oscillatory activity. Furthermore, the analyses are predominantly descriptive and may be overly influenced by the modeling choices. Thus, the differentiation of functional vs pathological stats seems mostly speculative and not supported by additional experimental data, where new insights from the model analysis can, at least in part, be found in EEG data recordings of patients.

> *Thanks to the reviewer for pointing out this issue. We agree and, in the revised version of the manuscript, we changed the abstract and introduction to focus solely on an information dynamics description of the many rhythms emerging in the dynamical phase diagram of the present model. We will leave the discussion about high-frequency oscillations as one interesting case study in which we see that with Phi-ID, we can distinguish "in silico" similar high-frequency oscillations (gamma) by the information dynamics of different neuronal populations (excitatory and inhibitory), indicating a different functional role for such similar waves.*
>
> *This difference found in functional properties related to HFOs in our model, seems similar to current discussions in the neuroscience literature about pathological vs physiological high-frequency oscillations. Therefore, we have conserved a discussion about the possible use of this framework to address this issue [see new subsection Rhythms, informational properties and functions in Discussion section, and in the Limitations of our… section). However, a deep study to conserve or discard our hypothesis will be conducted in future work using actual human and rat EEG/LFP/ECoG data.*

Comment 2: In my view, there are two possible directions for improvement:

1) Re-evaluate the paper's main focus. It seems more that the authors want to provide a comprehensive understanding of the role of the modeling parameters in the emergence of different oscillatory dynamics

and their information processing capabilities. The role of high-frequency oscillations and their role on functional vs pathological brain states distinction can be only discussed at the end in a 'Future direction' section, but it's not highly introduced as the main focus of the paper.

2) Alternatively, streamline the initial sections to focus more narrowly on high-frequency oscillations and think of a possible application to EEG data (with open epilepsy datasets, maybe) where the findings on high-frequency oscillation apply or provide new insights on EEG data. Although the modeling approach offers a level of detail that EEG data alone cannot provide, the authors might consider analyses that establish correspondences between the two. This could involve examining the IID differences (such as redundancy, differentiation, etc.), for example in epilepsy versus control groups, as well as mutual information in both the time and frequency domains. The goal would be to determine if similar conclusions can be drawn from EEG data as those inferred from the model.

> *Thanks to the reviewer for suggesting these two possible directions for improvement of our manuscript. In the revised version of the manuscript, we considered the first direction and revised the entire manuscript accordingly. We focused on providing a comprehensive understanding of the role of modeling parameters in the emergence of different oscillatory dynamics and their information processing capabilities.*
>
> *We acknowledge the importance of the second direction and the potential application to EEG data, particularly in epilepsy research. While we did not incorporate experimental data analysis into the current revision, we will explore this possibility in future research endeavors. We aim to establish correspondences between the findings of our "in silico" study and EEG data to enhance the clinical relevance and applicability of our work.*
>
> *We appreciate the reviewer's feedback and we ensured that the revisions address the concerns raised.*

I can not recommend the paper for publication without this and the following substantial revisions.

Major comments:

Comment 3. The paper requires significant enhancement in terms of writing quality, grammar, and the clear exposition of concepts. The minor comments I provided address only a few issues, but a comprehensive review is needed for improvements.

> *In the revised version of the manuscript, we have taken into account the reviewer's feedback and significantly revised the grammar and writing style. Our aim was to enhance readability and clarify the exposition of concepts throughout the manuscript. We appreciate the reviewer's attention to detail and are committed to ensuring that the paper meets the highest standards of quality.*

Comment 4. Did the authors assess the robustness of their results by altering the E-I and I-E connections in the networks? For example, by replacing an I-E connection within a circle with a randomly positioned neuron outside of that circle.

> *Introducing E-I and I-E connections outside the circle does not alter the main phase diagram or the observation of different rhythms in membrane potential fluctuations, thus indicating the robustness of our results in this regard. However, as expected, these modifications to the E-I and I-E connections can influence the appearance of gamma waves. This is consistent with existing literature highlighting the primary role of local circuits, including local connections, in the emergence of gamma oscillations (see references below). Therefore, changes in local*

dynamics resulting from the introduction of such random connections would likely lead to changes in the resulting waves.

References:

- *Cardin, J. A. (2016). Snapshots of the brain in action: Local circuit operations through the lens of γ oscillations. Journal of Neuroscience, 36(41), 10496–10504.* [*https://doi.org/10.1523/JNEUROSCI.1021-16.2016*](https://doi.org/10.1523/JNEUROSCI.1021-16.2016)
- *Buzśaki, G., & Wang, X. J. (2012). Mechanisms of gamma oscillations. In Annual Review of Neuroscience (Vol. 35, pp. 203–225).* [*https://doi.org/10.1146/annurev-neuro-062111-150444*](https://doi.org/10.1146/annurev-neuro-062111-150444)
- *Fernandez-Ruiz, A., Sirota, A., Lopes-dos-Santos, V., & Dupret, D. (2023). Over and above frequency: Gamma oscillations as units of neural circuit operations. Neuron, 111(7), 936–953.* [*https://doi.org/10.1016/j.neuron.2023.02.026*](https://doi.org/10.1016/j.neuron.2023.02.026)

To demonstrate this point, we modified our topology by randomly reconnecting an E-I or I-E link with a 0.2 probability to a neuron selected randomly across the network. Simulations were conducted within the same interval of mu and tau_rec but with reduced resolution (20x20) and fewer timesteps to enhance computational efficiency.

Our results indicate that the phase diagram remains unchanged (see Figure 1 below), and we still observe the emergence of waves. However, the specific region where high-gamma rhythm emerges varies depending on the topological details of the network (Figure 2 below).

We noticed that the region exhibiting high-gamma waves in E without corresponding emergence in I nearly disappears. Additionally, the region identified as having high-gamma waves with the worst informational properties remains present, albeit with a decrease in power density.

Given these findings, as our primary objective is to characterize the information dynamics across different regimes in the phase diagram of our model, introducing random connections does not significantly contribute to this goal. The phase diagram and the main behavior of our model remain unchanged. Furthermore, we are currently exploring more drastic modifications to the topology, including E-E and I-I interactions, for future studies. However, even with these modifications, much of the observed phenomenology remains unchanged.

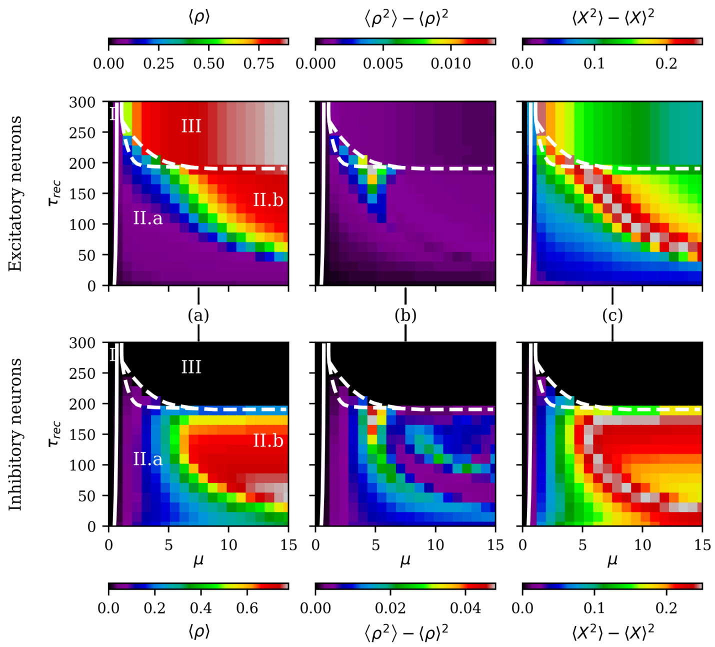

Figure 1. Phase diagram of EEG model with E-I/I-E reconnection probability of 0.2. The grid size used was 20x20. The main details of the phase diagram do not change in comparison with the original topology presented in our manuscript.

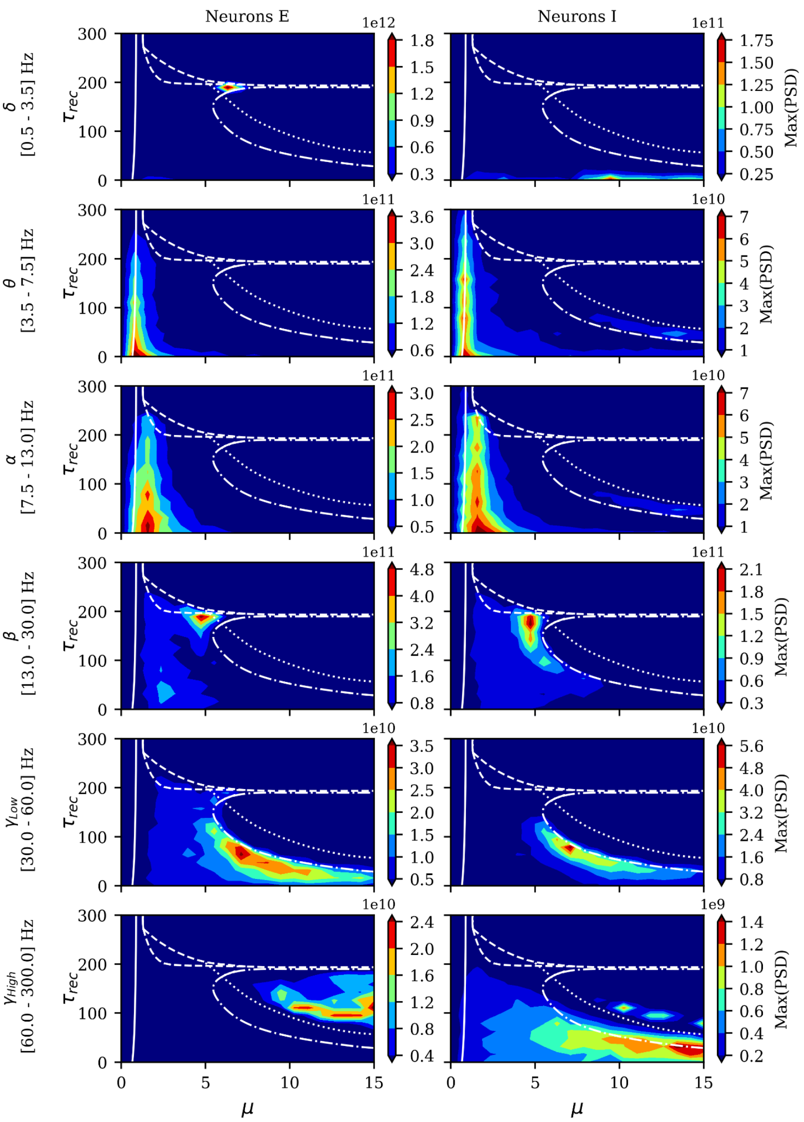

Figure 2. Maximum power density in different frequency bands across the phase diagram. Besides delta and high-gamma, the other rhythms were not affected by the reconnection of E-I and I-E links. The higher sensibility of High-Gamma to local topology details is in agreement with the literature about the local nature of these waves.

To address this and other points relative to the robustness of our model, we now include a new subsection "Model choices and topological details of the network" in the Discussion section.

Comment 5. In Figure 6, do the white areas on the phase diagram represent regions driven solely by noise, without a distinct PSD maximum? A brief explanation in the figure caption would be helpful.

Thanks to the reviewer for pointing out this issue. We missed explaining the meaning of the white areas in Figure 6. These white areas are depicted with colors in the supplementary Figure S1, where the color codes were explained in the previous version of the manuscript. In Figure 6, the white areas correspond to waves whose maximum power spectral density (PSD) does not reach the threshold we used to indicate clear emergent waves, which was set at 10^11 for the excitatory population and 10^10 for the inhibitory population. We have modified the text in the caption of Figure 6 to clarify what the white areas represent.

Comment 6. Is the coexistence of low-frequency (delta-theta) and high-frequency (gamma) oscillations in the phase diagram related to the low-high frequency coupling often observed in EEG data? Could the model provide new insights in terms of information dynamics?

We appreciate the reviewer for raising these intriguing questions. In previous literature with this model (Pretel, 2022), there is an initial discussion about the coexistence of low-frequency and high-frequency oscillations and its potential relation to the low-high frequency coupling commonly observed in EEG data. We acknowledge the possibility of exploring this issue further under an information dynamics approach with real data in future work. In our revised version of the manuscript, we now address this point similarly to the high-frequency oscillation issue.

Specifically, we characterize the information dynamics in both excitatory and inhibitory populations when delta and beta waves dominate, as well as when waves coexist. We found that dominant delta waves exhibit a clear relationship with information transfer in the excitatory population, while dominant beta waves are associated with information transfer in inhibitory neurons. Additionally, the coexistence of waves shows a clear relationship with maximizing information storage in the excitatory population. A detailed discussion of these results is now included in the revised manuscript (see "Insights on waves emergence through information dynamics: " subsection in the Results section, and "Rhythms, informational properties and functions" in the Discussion section).

Comment 7. Do the authors have an explanation for the high differentiation information observed in region III of the phase diagram, which appears prominent only at specific ranges of u noise levels? (Refer to Figure 7 and mainly S7).

This is indeed an interesting observation that we did not specifically address in our article. In Region III of the phase diagram, inhibitory activity ceases, leading to a decoupling of excitatory neurons from each other due to the absence of EE interactions in our network. Consequently, no collective behavior emerges in this region, and thus the emergence of waves is not observed. Therefore, we considered exploring the behavior of the system in phase III as outside the scope of our current article. However, we can provide a solid explanation for the behavior noted by the reviewer.

In region III, where neurons do not interact, all information between present (t) and future (t+tau) states is of the "Unique" type (each element has only information about itself). This explains why we observe Differentiated Information as different from 0.

The decoupling of excitatory neurons allows us to explain the peak in Differentiation by studying the individual dynamics of a single neuron under varying noise levels. In Figure 3, we display the Differentiated measure observed in a group of 12 excitatory neurons (as shown in Figure 7 and S7 of our article) alongside the Time Delayed Mutual Information of a single neuron (x_i) simulated outside a network, driven only by noise.

We observed that the peak in information occurs at approximately the same noise value, indicating that Differentiation captures information related to individual neuronal dynamics, as expected from its association with "Unique" information atoms. This peak coincides with the knee (mu^{*}) of the Firing rate curve, which was localized using the Kneed python package. At this point, the neuron begins to become saturated by noise and fires at a more or less fixed firing rate.

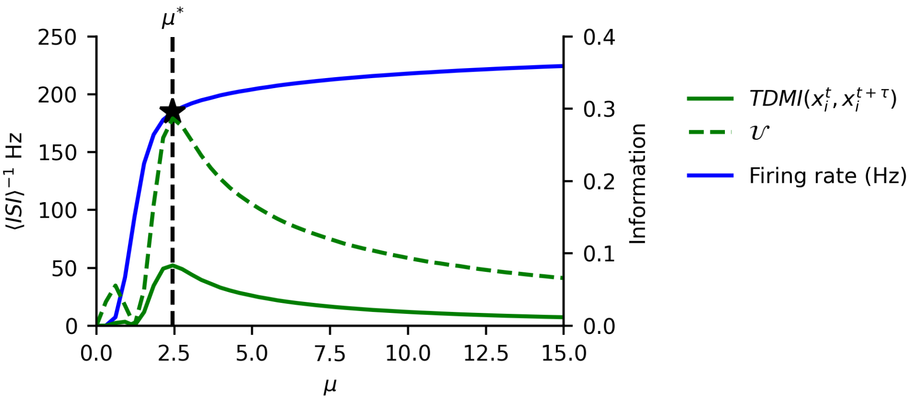

Figure 3. **Differentiated information peak**. Differentiated information (Green dashed line) computed for a group of 12 E neurons for tau_rec = 300 as shown in Figure 7 and S7 of the new revised article version. Time delayed mutual information for just one excitatory neuron state time series x_i (solid green line) and firing rate of this same neuron (solid blue line).

The knee of the firing rate curve, as shown in Figure 4 below, is related to a transition from an interspike interval distribution without a meaningful average (like a scale-free distribution) to a distribution with well-defined intervals average (neurons fire at more or less fixed time intervals). Below mu^{*}, spiking is less predictable, with neurons potentially experiencing large intervals without firing, reducing the informativeness of knowing the current state of a neuron (x_i[t]) about its future state (x_i[t+tau]). Conversely, for high noise levels, neurons asymptotically approach their maximum firing rate, leading to almost deterministic behavior where knowing the current state does not reduce uncertainty about future states. In the transition between these regimes, we observe the maximum informativeness, represented by the peak in Time-Delayed Mutual Information (TDMI). This explanation holds for different time delays (tau), where the computation of firing rate is related to an arbitrary time interval associated with the time delay.

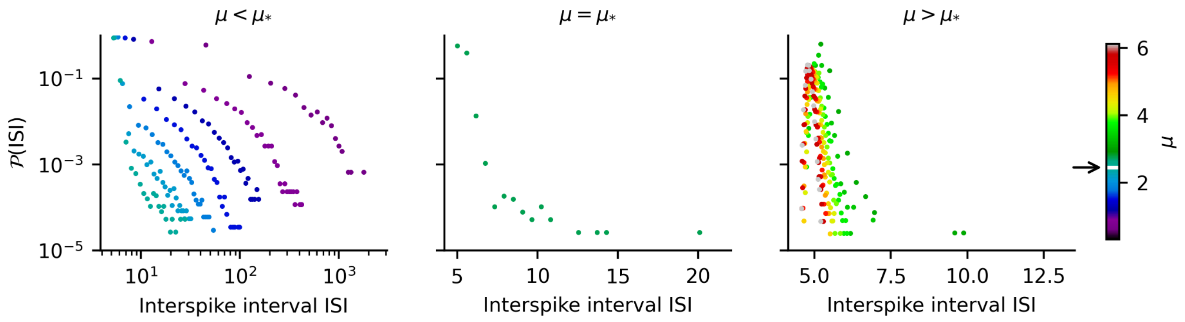

Figure 4. **Interspike interval probability distribution for an excitatory neuron driven only by noise**. For values below our mu^{*} (knee of the firing rate curve, peak of TDMI and Differentiation in Figure 3 above) we see a distribution with large variance. For values above mu^{*} *distribution has low variance and therefore a well defined average. Arrow indicates the color and value of mu^{*}.*

Given the substantial length of our main text and Supplementary Material, and particularly considering that Phase III falls outside the scope of our article due to its lack of emergent oscillations, we have chosen not to incorporate the discussion presented in this response into our manuscript.

Comment 8. The estimation of Φ-ID in continuous data, without relying on kNN methods, is possible (as shown in Luppi, Mediano et al., 2022, Nature Neuroscience, and Mediano et al., 2021, ArXiv:2109.13186). Why didn't the authors apply these measures to analyze average membrane

potential (LFP-like) in continuous data? This could be discussed in the 'Limitations of our analysis' section."

We appreciate the reviewer for bringing up this point. In our model, we have individual neuron state information, which is already discrete and boolean. Thus, we considered simpler and more informative to choose this approach to capture the local information dynamics.

In the revised version, we acknowledge that using continuous data methods, as discussed in Luppi, Mediano et al., 2022 and Mediano et al., 2021, could be beneficial for analyzing average membrane potential (LFP-like) data in experimental settings. However, we believe that employing methods such as the gaussian estimator mentioned by the reviewer for analyzing continuous data would be more suitable for future works, where we will explore both model and experimental data to test our hypothesis related to informational properties related to different rhythms.

In our new version of the manuscript, we already acknowledge that using boolean data to connect with wave emergence is not feasible with most common recording techniques. However, in the revised version, we also discuss the possibility of using new high spatial and time resolution techniques such as neuropixel probes to obtain discrete data like the one used in our current analysis. This is proposed as potential future directions of research to compare this model with real systems.

Comment 9. In the 'Limitations of our analysis' section, the authors should include references related to pathological HFOs and the less rich information dynamics and mutual information.

We appreciate the reviewer's suggestion. In the revised version of the manuscript, we have included several references that study pathological high-frequency oscillations (HFOs) in actual brains. Although various works use entropy-based methods to identify EEG signals related to epilepsy, to our knowledge, no existing references have explicitly linked such waves with less rich information dynamics. For example, a recently published book on intracranial EEG (Section 4, Chapter 23, Frauscher, 2023) discusses approaches and challenges to separate pathologic from physiologic HFOs. The authors include classical strategies such as measuring amplitude, duration, and spectral frequency, and also other promising strategies like utilizing evoked responses to distinguish between the two. However, they do not comment on informational-like analysis of neuronal activity time series for the same goal. Therefore, we consider that this kind of informational approach is still poorly explored. Through our work, we aim to motivate research in this direction among experimental groups. This issue is now discussed in the revised version of the manuscript in the discussion section.

Reference:

- Acharya, U. R., Fujita, H., Sudarshan, V. K., Bhat, S., & Koh, J. E. W. (2015). Application of entropies for automated diagnosis of epilepsy using EEG signals: A review. *Knowledge-Based Systems*, *88*, 85–96. https://doi.org/10.1016/j.knosys.2015.08.004
- Kbah, S. N. S., Al-Qazzaz, N. K., Jaafer, S. H., & Sabir, M. K. (2022). Epileptic EEG activity detection for children using entropy-based biomarkers. *Neuroscience Informatics*, *2*(4), 100101. https://doi.org/10.1016/j.neuri.2022.100101
- Travnicek, V., Klimes, P., Cimbalnik, J., Halamek, J., Jurak, P., Brinkmann, B., Balzekas, I., Abdallah, C., Dubeau, F., Frauscher, B., Worrell, G., & Brazdil, M. (2023). Relative entropy is an easy-to-use invasive electroencephalographic biomarker of the epileptogenic zone. *Epilepsia*, *64*(4), 962–972. https://doi.org/10.1111/epi.17539
- Frauscher, B., & Gotman, J. (2023). *How Can I Disentangle Physiological and Pathological High-Frequency Oscillations?* (pp. 377–388). https://doi.org/10.1007/978-3-031-20910-9_24

Comment 10. All elements depicted in the figures should be explained in their captions. For example, the significance of the black arrows in Figure 6.

Thanks to the reviewer for highlighting this issue in the previous version of the manuscript. We have carefully revised the manuscript and ensured that all elements illustrated in the figures are now thoroughly explained in their captions. Specifically, in Figure 6, we have added an explanation of the significance of the black arrows..

Comment 11. The supplementary material contains subsection 2.1, 'Excitatory and inhibitory activity', and Figure S5, which appear to be exact duplicates of content from the main text.

We apologize for this oversight. In the revised manuscript, we have corrected the figures and ensured that there are no duplicates between the main text and the supplementary material.

Minor comments:

Commnet 12. In the following I reported some of the issues with the writing. Please revise the all manuscript since it was impossible to track all grammar and syntax problems.

We would like to express our sincere gratitude to the reviewer for their thorough and detailed revision of our manuscript. Your insightful comments and suggestions have been invaluable in enhancing the clarity, coherence, and overall quality of our work. We have carefully addressed each point raised, ensuring that the necessary corrections and improvements have been made throughout the text. Your guidance has significantly contributed to the refinement of our research, and we deeply appreciate the time and effort you have dedicated to this review. Thank you for helping us to present our findings in a more effective and comprehensible manner.

The title point to EEG brain waves, the paper use only a model maybe change to -EEG neural network model

We changed the title and within the main text to address the fact that we are only working with a model by using the phrase "in silico", as commonly done in computational neuroscience literature.

Abstract
- NN ? Does it mean Neural network ?
- integrate-and-fire
- “However, in regions where high gamma … we observe generally lower ..”

Author summary:
-“i.e. the recording of ..”
-“where EEG-like signals emerge within a neural population”
-“Through this approach , we explore ...”

Improve writing of the Abstract and Author summary

We have revised both sections to align with the adjusted focus of our article, in accordance with your suggestion in Comment 2. The new abstract and author summary now better reflect the main emphasis of our research and provide a clearer overview of the objectives and findings presented in the paper. We appreciate your feedback and believe that these revisions will significantly improve the readability and impact of our manuscript.

Line 9: is loosely
Line 10: relaxation
Line 82- PID framework is not restricted to only 3 sources. Also PID is multivariate the difference with IID is that PID the does not allow to decompose multiple targets.

We correct the sentences following the indications of the reviewer.

Be consistent with the verb tense in the rest of the Introduction

Line 98: “which has not been explored ..”
Line 145: “which provides a”
Line 154 “it can be applied to decompose”

In general I find “give us” too formal.

Line 223: “expressed”
Line 251-254 split sentence
Line 258 “in inducing” ?
Line 261 “Using a simplified approach”
Line 266: “This model aims to capture the essentials of the cerebral cortex
where it is reported that excitatory neurons occur almost four times more than inhibitory ones.”
Line 268: “To couple”
Line 368: “.., making the observation of active information dynamics between distant neurons less probable;”
Line 335: Wrong Figure 3a, maybe Figure 4a ??
Line 384: “attention on Γ waves”. Is Γ symbol introduced already?
Line 388: Finally, we focus (remove and)
Line 689 write always High or H
Figure 6. colored stars ? not starts
Wrong figure reference in Supplementary material. Many figures show Figure ??

We appreciate the reviewer's valuable feedback, and we have made sure that our revisions address the concerns raised. Thank you again for your corrections, which have been instrumental in refining and improving our manuscript.

DATA AND CODE AVAILABILITY

Reviewer #1: No: I did not find in the manuscript the Data and Code availability section.

Reviewer #2: No: The authors did not provide the code for the modeling and the analysis.

We now include a Data and Code availability section, where we provide the link to a GitHub repository containing the code for both the modeling and the PhiID analysis.
