# Supplementary material for: Information dynamics of in silico EEG Brain Waves: Insights into oscillations and functions
Source: PLoS Comput Biol. 2024 Sep 5;20(9):e1012369. doi: 10.1371/journal.pcbi.1012369 (PMC11407780; doi:10.1371/journal.pcbi.1012369)
Supplement: Attachment — Submitted filename: Letter_to_Reviewers_Second_round.pdf [file pcbi.1012369.s005.pdf]

Reviewer #1: The reviewed manuscript has significantly clarified several open issues that I raised during the first review. However, there are a few minor points which are not clear. I think the final manuscript should try to clarify the following points.

1- INTRODUCTION

a- There is an imprecision about the definition of HFOs. There is no such thing as “HFOs in the γ band (30-150 Hz)”. Normally HFO are defined above approximately 80 Hz. To avoid confusion, I would stick to the definitions HFOs given by this paper:
https://www.nature.com/articles/s41467-020-18975-8
See refs 3 and 4 in the above paper for example: The definition of gamma band activity is also not precise. Studies investigating gamma-band neural activity are often considering two types of neural modulations. A narrow-band low-gamma oscillations in the 30–80 Hz range during sensory stimulation (Ray and Maunsell, 2010) and cognitive processes, such as attention (Fries et al., 2001; Bosman et al., 2012) and working memory (Pesaran et al., 2002). And a second type of high-gamma range (from 60 to 150 Hz) activity is observed in invasive (e.g., Brovelli et al., 2005; Crone et al., 2006; Jerbi et al., 2009) and noninvasive (MEG) studies. This second type of gamma band activity is broadband rather than purely oscillatory. I think it would be better to specify which type of gamma activity the manuscript is focusing on and whether it is possible to discern the two.

Thank you for raising this important point. As shown in the Supplementary Information, the high-frequency oscillations in Phase II.b of our model are consistently of the narrow band type, with a well-defined narrow peak in the power spectrum at high frequency. Therefore, following the reviewer's suggestions, we will adopt the definitions presented in the article by Arnulfo et al. (2020) and refer to these as fast-gamma oscillations (narrow band oscillations between 90-150 Hz) instead of high-gamma oscillations. We acknowledge that using the term "high" could be confused with high-gamma activity (HGA), which, as the reviewer pointed out, includes more than purely oscillatory phenomena.

In the revised version, we also clarify the distinction between high-frequency "activity" (which encompasses all brain activity above 80 Hz) and high-frequency "oscillations" (which refer to any discrete EEG events of at least four oscillations). Our model specifically explores the latter.

However, we recognize that there is often a lack of consensus in the literature regarding the ranges and classifications of high-frequency bands. For instance, the article suggested by the reviewer illustrates this point: in the first paragraph of the introduction, it classifies high-gamma activity (HGA) from 40-150 Hz according to Lachaux et al. (2012), but also uses the range 80-200 Hz as per Ray and Maunsell (2011), which aligns more closely with the literature we initially referenced for high-gamma oscillations.

b- The introduction still contains the presentation of several results. I would move these paragraphs from the introduction to the results section. Lines 143 - 167 for example.

Following the suggestions of both reviewers, we have deleted some introductory paragraphs and phrases, as they were redundant and already presented in detail in the results and discussion sections.

2- RESULTS

If the labeling of the different oscillatory bands is modified according to the novel nomenclature (first point above), I would suggest revising the figures.

Exactly, we have changed all the labels to "fast gamma" instead of "high gamma" to refer specifically to narrow band oscillations, as suggested by the reviewer's recommended literature.

3- CODE

The data and code underlying the findings described in their manuscript should be fully available without restriction. The repository is very limited and it could contain some additional script for the analysis and visualization of the resutls. Maybe some example notebook could help. I leave this point to the authors.

The Zenodo repository already contains a notebook and all processed data (2.1 GB data files) for computing the results and generating all figures presented in the manuscript.

Now, following the reviewer's suggestion, we have included in the GitHub repository an example notebook for running simulations with different values of noise level (mu) and recovery time (tau_{rec}). We also included a notebook for visualizing raw data: raster plots of individual neurons and average membrane potential for each E/I neuron group, as shown in Figure 1.

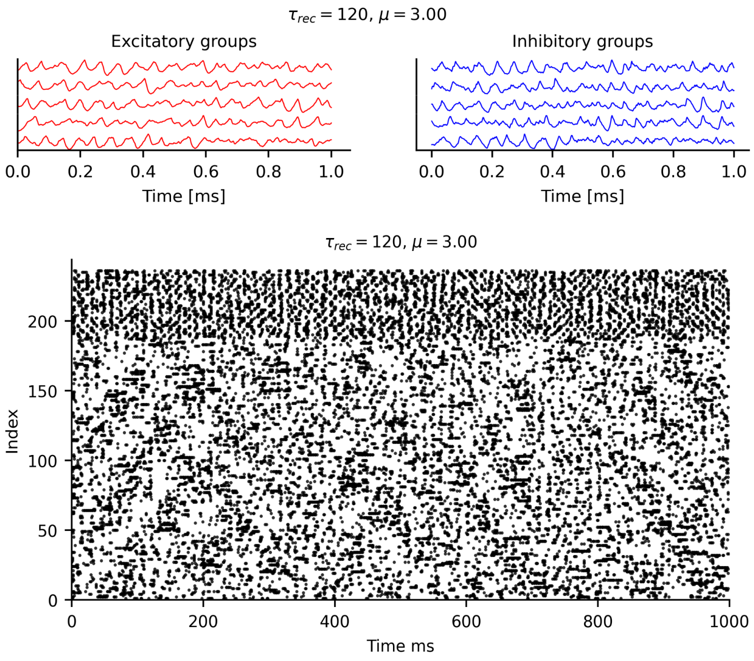

Figure 1. Raster plot and membrane potential signal for neurons groups generated with the example notebook, simulation for specific value of tau_rec and mu.

We also included a notebook for selecting a group of neurons from the boolean time series file and analyzing the Phi-ID, presenting the values for the 16 atoms of each possible partition in a .csv file. The new version of the repository also contains an example notebook using Python to read this output and compute all information measures used in the article for an example file.

With these scripts, any reader can run their own simulations with different model parameters and implement their analyses in the same fashion as our article.

The raw data for each 50 x 50 phase diagram is too large to make fully available easily. The reviewer should note that we ran simulations for three different regions of 50 (mu) values x 50 (tau_{rec}) values: one for the full diagram (mu = [0,15]) and (tau_{rec} = [0,300]), one for the high activity region where HFOs emerge, and one to cover the region in detail where metastability occurs and to explore the peaks in beta and delta waves. For each point (mu, tau_{rec}), we computed and saved 10 time series of "average membrane potential" (one per each excitatory and inhibitory neuron group, 5 E groups, and 5 I groups), saving (10^7) time steps, and also saving (N = 245) neurons' raster data (boolean time series) of (2 x 10^5) time points (after binning using 4 ms time windows). This makes the total size of raw data for the three diagrams more than 2 TB. Given the size, we consider it not adequate to make it fully available, considering that anyone interested could compute it using the codes available in our repository.

Reviewer #1:
Minor revision
The authors satisfactorily addressed all the revision issues. I can recommend the manuscript for publication after the following minor revisions:

1- INTRODUCTION
The “Introduction” is still difficult to read. I suggest reducing the discussion of the results at the end of the section. Only mention one or two key findings with a few sentences.

Following the suggestions of both reviewers, we have deleted some introductory paragraphs and phrases related to the results, as they are already presented in full detail in the Results and Discussion sections. Additionally, we have reordered the presentation of ideas to make the Introduction more concise and easier to read.

2-DISCUSSION
In the Discussion for the role of different frequency bands on information storage in neural data (for example, low frequency and high information storage) you might cite the paper: Information theoretic evidence for layer- and frequency-specific changes in cortical information processing under anesthesia .Pinzuti E, Wollstadt P, Tüscher O, Wibral M (2023) Information theoretic evidence for layer and frequency-specific changes in cortical information processing under anesthesia. PLOS Computational Biology 19(1):e1010380.
https://doi.org/10.1371/journal.pcbi.1010380

We want to thank the reviewer for pointing out this article. It is indeed interesting and highly pertinent to our discussion on information storage. We apologize for not being aware of it earlier. In the new version of our manuscript, we refer to this article in our discussion.
