# Supplementary material for: Information dynamics of in silico EEG Brain Waves: Insights into oscillations and functions
Source: PLoS Comput Biol. 2024 Sep 5;20(9):e1012369. doi: 10.1371/journal.pcbi.1012369 (PMC11407780; doi:10.1371/journal.pcbi.1012369)
Supplement: Attachment — Submitted filename: Minor_revision_plosComp2024.doc [file pcbi.1012369.s004.doc]

**Minor revision**

The authors satisfactorily addressed all the revision issues. I can recommend the manuscript for publication after the following minor revisions:

-The “Introduction” is still difficult to read. I suggest reducing the discussion of the results at the end of the section. Only mention one or two key findings with a few sentences.

-In the Discussion for the role of different frequency bands on information storage in neural data (for example, low frequency and high information storage) you might cite the paper: [Information theoretic evidence for layer- and frequency-specific changes in cortical information processing under anesthesia](https://journals.plos.org/ploscompbiol/article?id=10.1371/journal.pcbi.1010380)**.**Pinzuti E, Wollstadt P, Tüscher O, Wibral M (2023) Information theoretic evidence for layer- and frequency-specific changes in cortical information processing under anesthesia. PLOS Computational Biology 19(1):e1010380. <https://doi.org/10.1371/journal.pcbi.1010380>
